# Impact of alignment algorithm on the estimation of pairwise genetic similarity of porcine reproductive and respiratory syndrome virus (PRRSV)

**DOI:** 10.1186/s12917-019-1890-0

**Published:** 2019-05-08

**Authors:** Marie-Ève Lambert, Julie Arsenault, Benjamin Delisle, Pascal Audet, Zvonimir Poljak, Sylvie D’Allaire

**Affiliations:** 1 Laboratoire d’épidémiologie et de médecine porcine (LEMP), Faculty of Veterinary Medicine, Université de Montréal, St. Hyacinthe, Quebec, Canada; 2 Swine and Poultry Infectious Diseases Research Center (CRIPA), Faculty of Veterinary Medicine, Université de Montréal, St. Hyacinthe, Quebec, Canada; 3 Department of Population Medicine, Ontario Veterinary College, University of Guelph, Guelph, Ontario Canada

**Keywords:** Porcine reproductive and respiratory syndrome virus, PRRS, Alignment algorithm, Sequence, Genetic similarity

## Abstract

**Background:**

Porcine reproductive and respiratory syndrome (PRRS) is a major threat to the swine industry. It is caused by the PRRS virus (PRRSV). Determination and comparison of the nucleotide sequences of PRRSV strains provides useful information in support of control initiatives or epidemiological studies on transmission patterns. The alignment of sequences is the first step in analyzing sequence data, with multiple algorithms being available, but little is known on the impact of this methodological choice. Here, a study was conducted to evaluate the impact of different alignment algorithms on the resulting aligned sequence dataset and on practical issues when applied to a large field database of PRRSV open reading frame (ORF) 5 sequences collected in Quebec, Canada, from 2010 to 2014. Five multiple sequence alignment programs were compared: Clustal W, Clustal Omega, Muscle, T-Coffee and MAFFT.

**Results:**

The resulting alignments showed very similar results in terms of average pairwise genetic similarity, proportion of pairwise comparisons having ≥97.5% genetic similarity and sum of pairs (SP) score, except for T-Coffee where increased length of aligned datasets as well as limitation to handle large datasets were observed.

**Conclusions:**

Based on efficiency at minimizing the number of gaps in different dataset sizes with default open gap values as well as the capability to handle a large number of sequences in a timely manner, the use of Clustal Omega might be recommended for the management of PRRSV extensive database for both research and surveillance purposes.

## Background

Porcine reproductive and respiratory syndrome virus (PRRSV) infection has a major economic impact on the swine production with annual cost estimated at $664 M for the US industry [1]. The virus causes reproductive failure as well as respiratory problems, impaired growth performance and increased mortality in growing pigs [2]. The important heterogeneity observed among North American PRRSV strains, combined with the absence of complete cross-protection following infection with heterologous PRRSV strains complicates disease management [3, 4]. Prevention of the disease mostly relies on limiting between-herd transmission that could occur through several direct and indirect pathways. In that regards, the genetic diversity in PRRSV can be used to support epidemiological investigations of a likely common source of infection or transmission events between herds in a research, surveillance or control context. A pairwise nucleotide sequence similarity ≥97.5% is the threshold often used to indicate if two sequences are considered similar and likely to originate from a same source [5, 6]. This threshold is also used into molecular-based interactive tools for field investigations on sources of contamination [7]. These tools are used to generate hypotheses about how a specific herd got infected which can orient the implementation of specific preventive measures to avoid further introduction and spread of the virus.

The alignment of sequences is a prerequisite for the estimation of genetic distances between pairs of sequences. Several algorithms to align sequences are available but they differ in terms of computing approaches. Dynamic programming is an exact method evaluating each possible alignment to determine the best solution. Unfortunately, it is too cumbersome to be run for more than a few sequences. Thus, heuristic methods, progressive or iterative, are preferably used to manage large sequence databases; they progressively incorporate pairwise alignments into multiple alignment which considerably decrease computing time [8]. Also, global or local methods can be chosen according to how similarities are maximized throughout the alignment process; global methods consider the entire sequence length whereas local ones focus on highly homologous areas of the gene [9]. Finally, algorithms can be distinguished by the number of sequences considered in the alignment process and the purpose of analysis. A particular sequence can be aligned to each sequence found in a database to find the most similar one using a pairwise sequence alignment, or a large group of sequences can be aligned simultaneously with multiple sequence alignment to better take into account genetic evolution [10].

For most algorithms, the alignment process generally arranges gene sequences one over the other to maximize identical matches of nucleotides between sequences [8]. Generally, algorithms try to optimize an objective function minimizing mismatches and gaps. In fact, gaps can be inserted during the alignment process if deletion or insertion sites are detected by the algorithm, so that sites with identical nucleotides align together. Using large penalties (cost) for opening a gap and a much smaller one for extending it result in programs adding fewer and/or shorter gaps [11]. Due to differences in objective functions or approaches used to optimize them, as well as several other parameter settings such as gap penalty, variations can be observed in final alignments obtained from different algorithms, sometimes leading to differences in inferred phylogenetic trees [12].

Even if preliminary evaluation of several alignment algorithms or gap penalty settings on biological datasets is often suggested, this is rarely done, and studies on PRRSV are not an exception [13–15]. In fact, the choice of the best algorithm is not a straightforward task when using field data, since no reference alignment is available contrarily to simulated dataset or reference alignment based on the three-dimensional superposition of the proteins [16, 17]. Some studies have compared algorithms for highly divergent sequences belonging to different families of genes, or based on a collection of known protein genes (e.g. ribosomal 16S or 23S subunit) across many species and showing important variation of sequence length [16–18]. However, studies on PRRSV North American genotype generally focus on relationships among sequences from a single viral gene (ORF5) that is expected to have considerably less diversity (≤25%) and to be relatively well conserved in length [19–21], which are two characteristics reported to influence the alignment process [11]. Although one could expect that default parameters set to align distantly related sequences should also work on less complex dataset, it has been suggested that these parameters should be evaluated on biological sequence datasets used in a specific context [11, 22]. Also, it has been recommended to test simultaneously different algorithms, particularly on large-scale phylogenetic studies [23]. The rationale being that congruent results among several techniques should give better support to the accuracy of the final alignment [24]. This will also provide useful information on the comparability of results from PRRSV diversity studies based on different alignment programs.

This study was conducted to evaluate the impact of the alignment algorithm on the resulting aligned sequence dataset as well as on practical issues such as the capability to handle large dataset in a timely manner when applied to a large database of PRRSV ORF5 sequences used for molecular-based epidemiological studies and surveillance program.

## Methods

### Data collection and study population

The PRRSV ORF5 sequence database of the Laboratoire d’épidémiologie et de médecine porcine (LEMP) of the Université de Montréal was used for the study. This database comprises sequences from field samples submitted by veterinarians to different laboratories on a voluntary basis as part of their herd surveillance or control programs. Since January 2010, a sharing agreement with 97% of all Quebec swine veterinarians have ensured that all PRRSV ORF5 sequences obtained from their field submissions to the veterinary diagnostic laboratory of the Université de Montréal or to two other private laboratories were automatically transferred to the LEMP. All sequences gathered between January 1st 2010 and December 31st 2014 inclusively (*n* = 2383), with the exception of one 606 base pair (bp) sequence, were used for the evaluation of alignment algorithms. This latter sequence was removed to ensure a balance of sequence length (600, 603 bp) in further replicates.

### Laboratory analyses

RT-PCR and sequencing of the gene ORF5 coding for the major envelope protein GP5 was performed on all samples to identify a PRRSV sequence. Approximately 55% of sequences gathered in the LEMP sequence database between 2010 and 2014 were submitted to the Veterinary Diagnostic Laboratory of the Université de Montréal. RNA was first extracted from serum or tissues (e.g. lungs, tonsils) with different extraction kits according to manufacturer’s instructions. RT-PCR was performed using Qiagen OneStep RT-PCR Kit using various primers. Prior to ORF5 sequencing, purification of PCR products was done using EZNA Cycle Pure Kit (Omega Bio-tek inc, Norcross, Georgia, US). Afterwards, both strands of PCR amplicons were sequenced using the same RT-PCR primers with BigDye terminator on ABI Genetic analyzer (Applied Biosystems Canada, Streetsville, Ontario, Canada). The remaining sequences were obtained from private diagnostic laboratories which have used their routine protocols.

### Detection of recombinant sequences

Detection of recombinant sequences was carried out by doing an exploratory scan for mosaic signals using default detection methods implemented into Recombination Detection Program (RDP) version 4.76 [25]. Primary scan was performed using only RDP, Geneconv and MaxChi. These latter methods were then used in addition to Bootscan, SisScan, Chimaera and 3-Seq for secondary scan. For each detection method, default parameter settings were used. Sequences identified by at least one primary method considering a 0.05 *p*-value using a Bonferroni correction for multiple testing were considered as significant recombinants.

### Alignment algorithms

#### Selection

Considering that a high level of similarity was expected over the entire ORF5 gene and that the overall objective was to manage a large PRRSV sequence database, only global multiple sequence alignment methods available in freeware were considered for selection. Five algorithms were selected considering their accuracy and popularity: Clustal W v.2.1 [26], Clustal Omega v.1.2.0 [27], Muscle v.3.8.31 [28], T-Coffee v. 11,00,8cbe486 [29] and MAFFT v.7.215 [18].

#### Parameter settings – other than open gap penalty value

When possible, options were set to obtain the maximal accuracy reachable by the algorithms according to the user manual provided by their authors. For Clustal W, sequences were aligned in pairs using dynamic programming to generate a DNA weight matrix using International Union of Biochemistry (IUB) scoring matrix (used in BESTFIT). Scores were then converted into distances and used to build a neighbor-joining guide tree (option Clustering = NJ). Iteration refinements were performed throughout the progressive approach (option Iteration = Tree). Default settings were used for Clustal Omega except for the use of full distance matrix in guide-tree calculation and iteration. Default settings were used in Muscle and T-Coffee. For MAFFT, G-INS-I was chosen based on highest accuracy and suitability for the study of sequences having similar lengths (MAFFT manual 2007-06-09, https://mafft.cbrc.jp/alignment/software/manual/manual.html). Needleman-Wunsch algorithm computed pairwise alignments (globalpair option) in combination with a maximum of 1000 cycles of iterative refinement or convergence of scoring alignment (option maxiterate = 1000). Default values were attributed to other parameters except for the open gap penalty value.

#### Parameter settings for open gap penalty value

**Fig. 1:**
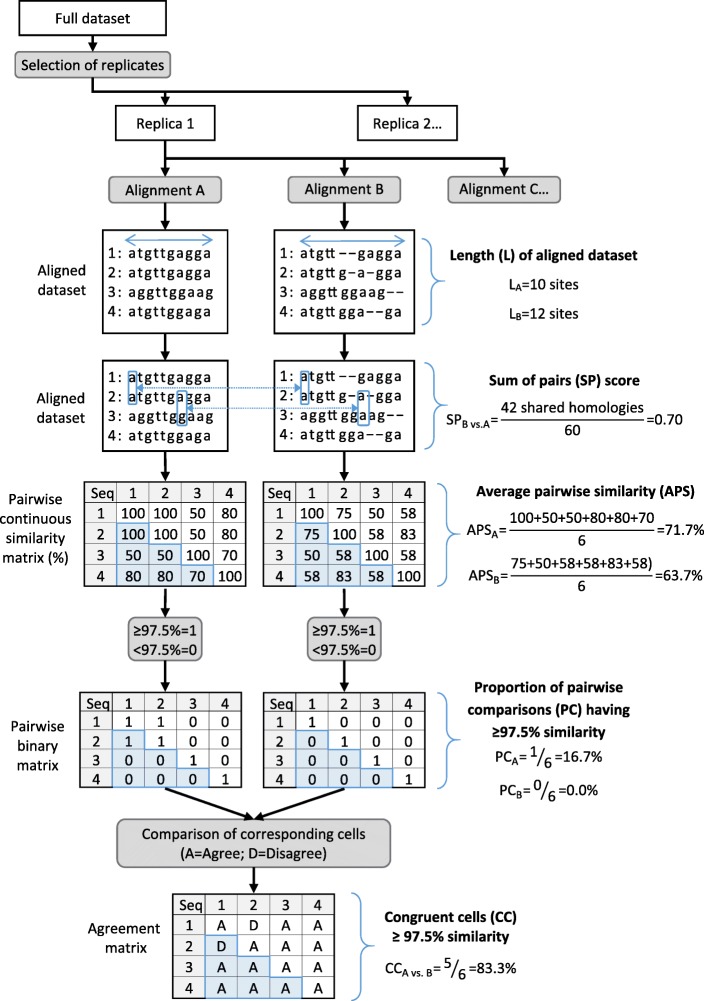
Operational workflow used for computations of analytical criteria. Analytical criteria results are pictured in blue whereas pairwise matrices represent intermediary steps involved in computations. Illustrated with a fictive dataset of 4 sequences aligned with 2 algorithms (A and B)

For each alignment algorithm, a sensitivity analysis was used to determine the open gap penalty parameter to be used for subsequent comparison of algorithms. The only exception was for Clustal Omega, for which the gap parameter is directly handled by the algorithm. Sequences including recombinants (*n* = 2383) were randomly selected without replacement to form replicates of different sizes: ten replicates of 238 sequences, five replicates of 476, two replicates of 1191 and one including all 2383 sequences. For each algorithm and replicate, alignment was attempted for 11 different open gap penalty values i.e. from baseline to upper limit by equal increment. The open gap penalty values were the following: Clustal W (0 to 100 by 10), MAFFT (0 to 10 by 1), Muscle (0 to − 1000 by − 100) and T-Coffee (0 to − 1000 by − 100). The impact of gap penalty value was evaluated according to three criteria: average pairwise genetic similarity, proportion of pairwise comparisons having ≥97.5% genetic similarity (Fig. 1), as well as the maximal number of gaps introduced per sequence. The open gap value from which a plateau was reached for the three criteria (i.e. minimum average pairwise similarity, minimum proportion of pairwise comparison with ≥97.5% genetic similarity, minimum number of gaps) was selected for further analyses based on visual assessment. Dataset sizes unable to run on all algorithms in less than 2 weeks were not considered for the choice of the open gap value.

#### Implementation

All alignments were run in a Linux environment (Ubuntu 14.04 LTS) on a Dell Precision T7610 workstation with 10 Intel Xeon Processor E5–2670 @ 2.5 GHz, 128 GB of RAM (DDR3) and a 2 TB HD. All computer resources were solely attributed to the alignment process.

### Selection and evaluation of comparison criteria

#### Analytical criteria

To compare performances of the five algorithms, two replicates of 1191 sequences were created from the dataset. A stratified random selection was used for sequence allocation to each replicate to ensure a similar proportion of sequences with 600 and 603 bp as well as recombinant sequences in each replicate.

The average pairwise genetic similarity, the proportion of pairwise comparisons having ≥97.5% genetic similarity and the length of final aligned dataset were computed for the two replicates for each algorithm using either SAS version 9.3 software (SAS Institute Inc., Cary, North Carolina, USA) or scripts written in Python. Characteristics of gap insertions, i.e. if they were introduced in singleton or triplets, were noted for each aligned dataset.

Two additional criteria were evaluated: the sum of pairs (SP) score and the percentage of congruent cells having ≥97.5% similarity. SP-score is a measure of accuracy defined as the proportion of shared homologies by estimated and reference alignments over the total number of homologies in the reference alignment [22]. Since the reference alignment was unknown in the current study, alignment from each algorithm was by turns considered as the reference, and the SP-score was used as a measure of agreement between algorithms. SP-score was computed using FastSP, an open-source executable written in Java available online [23]. The percentage of congruent cells having ≥97.5% similarity among algorithms was computed as follows. For each alignment, a pairwise similarity matrix was calculated and transformed into a binary matrix: 0 for < 97.5% similarity, 1 for ≥97.5%. For each combination of two alignment algorithms, the binary matrices were compared and the proportion of cells in agreement (having the same binary value) over total number of cells was computed. The operational flowchart used in computations of all analytical criteria is described in Fig. 1.

#### Technical criteria

Four technical criteria were also used to compare performance of algorithms, i.e. handling capability of large dataset, rapidity, multi-platform availability and management of IUB ambiguity symbol characters (symbols other than A, T, C and G). The first two criteria were evaluated for 10 replicates of 238 sequences, 5 of 476, 2 of 1191 and 1 of 2383. Results were averaged over replicates. Data were analyzed in SAS.

#### Sensitivity analysis on recombinant inclusion

All analyses described for the analytical criteria assessment were reconducted to evaluate the impact of recombinant sequences using the same two replicates of 1191 sequences, but without detected recombinants (*n* = 1183).

## Results

**Fig. 2:**
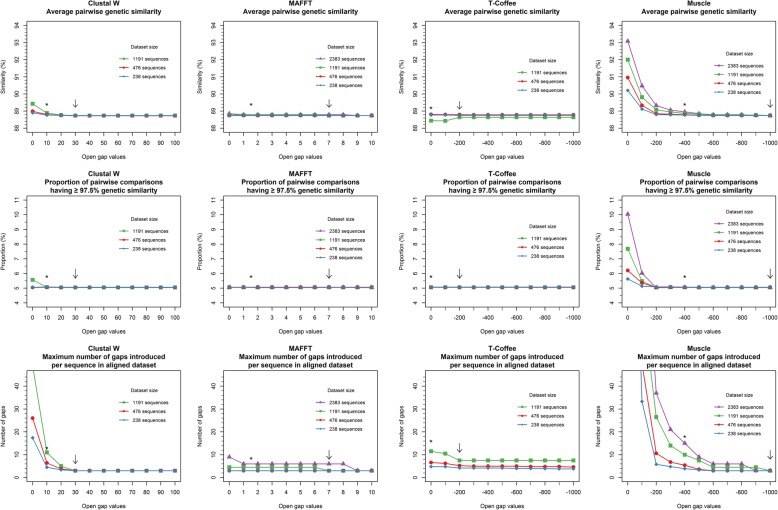
Impact of open gap penalty value on average pairwise similarity, proportion of pairwise comparison having ≥97.5% genetic similarity and maximal number of gaps introduced per sequence for Clustal W, MAFFT, T-Coffee and Muscle. The different statistics were computed on each aligned dataset. Results obtained for dataset sizes of 238, 476 and 1191 were averaged over 10, 5 and 2 replicates, respectively. Results for the 2383 dataset are shown only for algorithms that generated results in less than two weeks. Recombinants were included in the datasets. Arrows indicate open gap penalty value selected for further analyses. *Default value of open gap penalty as defined by the algorithm user manual

Following the sensitivity analysis for the determination of open gap penalty values, the open gap penalty parameter was set at 30 for Clustal W, 7 for MAFFT, − 200 for T-Coffee, − 1000 for Muscle and default value for Clustal Omega. In general, the open gap penalty value had only a minimal impact on average pairwise similarity, proportion of pairwise comparisons having ≥97.5% similarity and maximal number of gaps per sequence for MAFFT and T-Coffee, but was more influential for Muscle and to a lesser extent for Clustal W (Fig. 2). The impact of the gap penalty value on the pairwise similarity and number of gap introduced tended to increase with dataset size, but convergence was obtained at approximately the same open gap penalty value whatever the size of the dataset. For each algorithm, a plateau was observed generally first (i.e. at lower gap penalty value) for the proportion of pairwise comparisons having ≥97.5%, followed by the average pairwise similarity and number of gaps. Once the plateau was reached for the three parameters, all algorithms converged to a similar number of gaps introduced (i.e. 3) for datasets with ≤1191 sequences, except for T-Coffee which introduced more gaps (up to 9 for one replicate of 1191 sequences).

A total of 17 recombinant sequences were identified within 12 distinct recombination events. In order to allow even number of recombinants (*n* = 8) in each replicate (*n* = 1191) formed to investigate analytical criteria, one 603 bp recombinant sequence was excluded. The evaluation of the analytical criteria revealed a high and very similar average pairwise genetic similarity across all algorithms and replicates, ranging from 88.28 to 88.84% (Table 1). The proportion of pairwise comparisons of sequences having ≥97.5% genetic similarity was also very similar, ≤0.25% variation between algorithms within replicates and a slightly larger variation (0.5%) between replicates for the same algorithm. The sequence length of aligned dataset differed according to algorithm. Whereas Clustal W, Muscle and Clustal Omega introduced the minimal number of gaps (*n* = 3) on 600 bp sequences to integrate them with the 603 bp sequences in the final alignment, MAFFT introduced 3 to 6 gaps and T-Coffee, 7 to 9 gaps depending on replicates. For Clustal W, Clustal Omega, MAFFT and Muscle algorithms, all gaps were introduced as triplets, representing the code frame shift. Most gaps (> 98%) introduced by T-Coffee were singletons. Based on the SP-score, more than 99.7% of all pair homologies were shared between each combination of two algorithms, with T-Coffee showing a slightly higher disagreement with all other algorithms. A similar finding was observed for the proportion of congruent cells (≥99.86%).

**Table 1:** Results on analytical criteria investigated in a comparative study on PRRSV sequence alignment algorithms^a^

Criterion	Algorithm				
	Clustal W	MAFFT	T-Coffee	Muscle	Clustal 0mega
1. Similarity: average pairwise genetic similarity (%) of aligned sequences within the dataset (mean ± standard deviation)					
Replicate 1 (1191 sequences)	88.77 ± 4.19	88.84 ± 4.17	88.71 ± 4.23	88.78 ± 4.19	88.78 ± 4.19
Replicate 2 (1191 sequences)	88.68 ± 4.11	88.69 ± 4.11	88.28 ± 4.31	88.69 ± 4.11	88.69 ± 4.11
2. Proportion of pairwise comparisons of sequences having ≥ 97.5% genetic similarity (%)					
Replicate 1 (1191 sequences)	5.17	5.17	5.19	5.17	5.17
Replicate 2 (1191 sequences)	4.91	4.91	4.66	4.91	4.91
3. Length of aligned dataset: number of sites per sequence in the aligned dataset					
Replicate 1 (1191 sequences)	603	606	607	603	603
Replicate 2 (1191 sequences)	603	603	609	603	603
4. Average sum of pairs (SP) score: proportion of shared homologies with reference alignment (%)^b^					
Clustal W as reference	–	99.93	99.74	99.91	99.94
MAFFT as reference	99.93	–	99.78	99.97	99.97
T-Coffee as reference	99.92	99.96	–	99.94	99.97
Muscle as reference	99.91	99.97	99.76	–	99.95
Clustal Omega as reference	99.94	99.97	99.78	99.95	–
*Average*	*99.92*	*99.95*	*99.76*	*99.94*	*99.95*
5. Congruent cells ≥ 97.5% similarity: proportion of cells between two pairwise similarity matrices having the same binary value (0: < 97.5%; 1: ≥97.5%) for genetic similarity^b^					
Clustal W as reference	–	100.00	99.86	99.99	99.99
MAFFT as reference	100.00	–	99.86	99.99	99.99
T-Coffee as reference	99.86	99.86	–	99.86	99.86
Muscle as reference	99.99	99.99	99.86	–	99.99
Clustal Omega as reference	99.99	99.99	99.86	99.99	–
*Average*	*99.96*	*99.96*	*99.86*	*99.95*	*99.95*

^a^The open gap penalties used was 30 for Clustal W, 7 for MAFFT, −200 for T-Coffee, −1000 for Muscle and default for Clustal Omega. The dataset included 2383 sequences collected in 2010–2014 divided in two replicates

^b^Average of 2 replicates of 1191 sequences

All algorithms were able to handle datasets of up to 1191 sequences, whereas only MAFFT, Muscle and Clustal Omega could process a 2383 sequence dataset in less than 2 weeks (Table 2). The rapidity mirrored the same tendency, as MAFFT, Muscle and Clustal Omega were the fastest, independently of dataset size, aligning sequences in less than 20 s on the smallest dataset (238 sequences) and less than 29 min on the largest dataset (2383 sequences). T-Coffee and Clustal W were generally very slow, varying from 13 min for the smallest dataset (238 sequences) to over 9–17 h for the largest dataset processed (1191 sequences). All algorithms were available on the Web, Windows and Linux platforms. MAFFT and T-Coffee managed a greater number of IUB ambiguity symbols, followed by Muscle and Clustal series.

**Table 2:** Results on technical criteria investigated in a comparative study on PRRSV sequence alignment algorithms^a^

Criterion	Algorithm				
	Clustal W	MAFFT	T-Coffee	Muscle	Clustal 0mega
1. Handling capability of large dataset: capacity to generate results in less than 2 weeks (yes/no)					
10 replicates of 238 sequences	yes	yes	yes	yes	yes
5 replicates of 476 sequences	yes	yes	yes	yes	yes
2 replicates of 1191 sequences	yes	yes	yes	yes	yes
Full dataset (2383 sequences)	no	yes	no	yes	yes
2. Rapidity: average time (minutes) necessary to align (Linux platform, 10 physical cores)					
10 replicates of 238 sequences	12.8	0.2	13.1	0.2	0.2
5 replicates of 476 sequences	57.1	1.0	56.1	0.7	0.4
2 replicates of 1191 sequences	1040.5	7.0	540.0	3.9	1.2
Full dataset (2383 sequences)	n/a	28.5	n/a	17.0	2.9
3. Multiplatform availability (yes/no)					
Web, Windows and Linux	yes	yes	yes	yes	yes
4. Management of IUB ambiguity symbol characters: ability to manage symbols other than A, T, C and G					
List of managed symbols	N	N, R, Y, W, S, K, M, D, V, H, B	N, R, Y, W, S, K, M, D, V, H, B	N, R, Y	N

^a^The open gap penalties used was 30 for Clustal W, 7 for MAFFT, −200 for T-Coffee, −1000 for Muscle and default for Clustal Omega. The dataset included 2383 sequences collected in 2010–2014

Two replicates of 1183 sequences were formed by removing the 16 recombinants from the initial two replicates of 1191 sequences. The exclusion of recombinant sequences had a very minor impact on the results. The average pairwise similarity and the proportion of pairwise comparisons having ≥97.5% similarity slightly increase when excluding recombinant sequences (Table 3). For these two criteria, the greater difference was observed for T-Coffee. The length of aligned dataset, SP-score and proportion of congruent cells were very similar regardless of the presence of recombinants for all algorithms except T-Coffee for which small differences were observed, and this was mainly associated with the second replicate.

**Table 3:** Differences in results for analytical criteria when excluding or not recombinants for the different algorithms^a^

Criterion	Algorithm				
	Clustal W	MAFFT	T-Coffee	Muscle	Clustal 0mega
1. Difference in similarity: average pairwise genetic similarity (%) of aligned sequences within the dataset (mean)					
Replicate 1	0.01	−0.05	0.01	0.01	0.01
Replicate 2	0.02	0.01	0.15	0.01	0.01
2. Difference in proportion of pairwise comparisons of sequences having ≥ 97.5% genetic similarity (%)					
Replicate 1	0.07	0.07	0.07	0.07	0.07
Replicate 2	0.05	0.05	0.27	0.05	0.04
3. Difference in length of aligned dataset: number of sites per sequence in the aligned dataset					
Replicate 1	0	−3	0	0	0
Replicate 2	0	0	−1	0	0
4. Difference in average sum of pairs (SP) score: proportion of shared homologies with reference alignment (%)^b^					
Clustal W as reference	–	0.01	0.04	0.01	0.01
MAFFT as reference	0.01	–	0.04	0.01	0.00
T-Coffee as reference	0.01	0.01	–	0.01	0.00
Muscle as reference	0.01	0.01	0.04	–	0.00
Clustal Omega as reference	0.01	0.00	0.04	0.00	–
*Average*	*0.01*	*0.01*	*0.04*	*0.01*	*0.01*
5. Difference in congruent cells ≥ 97.5% similarity: proportion of cells between two pairwise similarity matrices having the same binary value (0: < 97.5%; 1: ≥97.5%) for genetic similarity^b^					
Clustal W as reference	–	−0.01	0.11	0.00	0.00
MAFFT as reference	−0.01	–	0.11	0.01	0.00
T-Coffee as reference	0.11	0.11	–	0.11	0.11
Muscle as reference	0.00	0.01	0.11	–	0.00
Clustal Omega as reference	0.00	0.00	0.11	0.00	–
*Average*	*0.02*	*0.02*	*0.11*	*0.03*	*0.03*

^a^The open gap penalties used was 30 for Clustal W, 7 for MAFFT, −200 for T-Coffee, −1000 for Muscle and default for Clustal Omega. The five criteria presented in Table 1 for the two replicates including recombinants (Replicates 1 and 2, *n* = 1191) were re-evaluated for each replicate without recombinants (Replicates 1 and 2, *n* = 1183). Then, differences in results were computed (i.e. the result obtained with recombinant was subtracted from the result obtained without recombinant

^b^Average of 2 replicates of 1183 sequences

## Discussion

We investigated the impact of the choice of alignment algorithm when applied on a PRRSV North American genotype 2 sequence dataset. The dataset of 2383 sequences employed was rather homogenous in regards to both similarity (≥79.1% minimum pairwise similarity obtained with different algorithms and open gap settings) and sequence length (603, 600 bp) reflecting viral population field studies as opposed to benchmark datasets such as BAliBASE [17, 18]. A priori, the multiple sequence alignment did not appear to face specific hurdles, and good accuracy from most algorithms was to be expected. In this study, although it was not possible to determine which alignment algorithm was more accurate due to the absence of a reference alignment [23], we compared algorithms by quantifying the variation in genetic similarity, which is important for molecular epidemiology studies on PRRSV. Moreover, algorithms were compared from a practical perspective, namely for surveillance of PRRSV which requires timely analyses.

The sensitivity analysis on open gap values revealed differences in gap management for the algorithms evaluated. In the study, the gap value parameter was optimized to minimize the number of introduced gaps. This decision seemed biologically sound since the aligned sequences were from one gene with no non-coding DNA, and that fewer gap insertions usually gives better alignment accuracy [30]. Globally, Muscle and Clustal W were the most affected by variation of the open gap parameter value and inserted a large number of gaps especially when the penalty was low. The results therefore supported that empirical investigations should be conducted before using default open gap value on a large PRRSV dataset. For all algorithms, default values were inadequate to minimize the number of gaps introduced into the resulting alignment, particularly on the datasets with more than 1000 sequences. However, using default open gap values on smaller dataset (*n* = 238 or 476) had a negligible effect for Clustal W and Muscle, and practically no effect for MAFFT and T-Coffee.

For PRRSV field investigations and molecular epidemiology studies, a pairwise genetic similarity threshold (e.g. ≥97.5%) is often used to determine whether two herds have similar strains [5, 6]. Results showed that most algorithms provided highly similar results in terms of average pairwise similarity of sequences, both when using the ≥97.5% similarity threshold or on continuous scale, and thus the use of different algorithms should not significantly affect epidemiological conclusions. This is also supported by the SP-score, which revealed that almost all pair homologies were conserved from one aligned dataset to the others, even when gaps were introduced. T-Coffee seemed to behave differently compared to other algorithms and showed more variation between replicates.

PRRSV usually evolves through punctual mutations, but recombination events are also a part of the virus evolution [31–34]. The detection of recombinant sequences should be an important concern for molecular epidemiology study using classic phylogenetic analyses since the evolutionary histories of recombinants are not correctly taken into account by these methods [33, 35]. Since the recombinants are not necessarily identified before the alignment process, either because of the need of a timely analysis of sequences for surveillance purposes or considering that alignment of sequences is the first step in investigating the presence of recombinants, it was therefore advisable to determine their influence on alignment according to the algorithm used. As expected, the inclusion of recombinants led to a decrease in pairwise genetic similarity; however, the results were highly similar between algorithms. Moreover, the number of gaps introduced stayed stable no matter if recombinants were included or not with the exception of T-Coffee. From an end-user perspective, even if excluding recombinants from PRRSV sequence dataset is favorable before performing phylogenetic analyses, their presence at the initial alignment step does not seem to influence the behavior of most algorithms, at least when they represent a small proportion of the sequences as we observed in our field database. The reasons underlying the greater influence of recombinants on T-Coffee were not investigated in our study; however, as these differences were mostly seen in one replicate, they could have resulted from specific characteristics of the recombinants or the sub-datasets.

Finally, technical aspects showed major differences in speed and capability of handling large datasets. Since runtimes vary according to genetic diversity observed in datasets, number and length of sequences, as well as processor and memory allowed for computing alignments, results obtained from different studies are not directly comparable. Considering the current datasets, algorithm settings and computational resources, the ability to timely align large sequence datasets (2383 sequences) by Clustal Omega, MAFFT and Muscle is a significant advantage.

## Conclusion

The different algorithms compared for the analysis of a PRRSV ORF5 sequence dataset provided very similar alignments, but differed in their ability to handle large datasets. Results from most algorithms were not affected by the presence of recombinants detected in our field database. Our study also revealed that prior investigations to set open gap parameter are advisable, especially when used on more than 1000 sequences. Muscle and Clustal W inserted many gaps when the open gap parameter was left at default or near zero values. Based on the efficiency at minimizing the number of gaps on different dataset sizes with default open gap value, the congruency of several analytical criteria with other algorithms as well as the capability to handle a large number of sequences in a timely manner, Clustal Omega might be warranted to manage large PRRSV database for both research and ongoing disease surveillance purposes.

## Abbreviations

LEMP: Laboratoire d’épidémiologie et de médecine porcine
ORF: Open reading frame
PRRSV: Porcine reproductive and respiratory syndrome virus
